# Retroperitoneoscopic approach for urolithiasis treatment

**DOI:** 10.1590/S1677-5538.IBJU.2019.0099

**Published:** 2020-08-10

**Authors:** Jose Luis Bauza, Valentí Tubau, Javier Brugarolas, Luis Ladaria, Carlos Aliaga, Pedro Piza, Enrique Pieras

**Affiliations:** 1 Department of Urology Hospital Universitario Son Espases Palma de MallorcaIlles Balears Spain Department of Urology, Hospital Universitario Son Espases, Palma de Mallorca, Illes Balears, Spain

## Abstract

**Objective:**

To show the main indications of retroperitoneoscopy (RP) for the treatment of urolithiasis. The use of RP approach has been limited, being narrow working space the major issue to overcome (1), especially in non-expert hands. However, RP has the added advantages of no peritoneal contamination, a quick recovery of bowel function (2) and the possibility to use it in combination with other endourological techniques (3) and innovative technology.

**Materials and Methods:**

We performed a retrospective analysis of 22 patients treated by the retroperitoneoscopic approach due to urolithiasis disease between 2015-2017. Type of surgery, stone free rate (SFR), complications according to Clavien-Dindo classification and mean hospital stay were recorded. Radical and partial nephrectomy cases were excluded for the SFR calculation. Descriptive statistical analysis was done using SPSS v21.

**Results:**

Of the 22 patients treated by the retroperitoneoscopic approach, 9 underwent a ureterolithotomy, 4 underwent a nephrolithotomy, 8 were nephrectomies and 1 was a polar nephrectomy. In 3 cases we used the indocianine green fluorescence (ICG) to find avascular planes, reduce the bleeding, permitting enhanced visualization and reconstruction. In 3 cases an additional percutaneous approach was used, increasing the SFR chances. Eleven of thirteen (84.6%) patients were stone free following the procedure. Tree complications were recorded, two Clavien II and one Clavien III complications. Mean hospital stay was 4 days.

**Conclusions:**

Retroperitoneoscopic approach is a good alternative for the treatment of large impacted ureteral stones, large pielic stones and for non-functional kidney removal due to stone disease. In expert hands, it can be safely used with a good SFR. The combination with ICG or other endourological techniques is feasible, allowing higher SFR.
